# Familial White–Sutton Syndrome Caused by a Pathogenic POGZ p.Arg508* Variant: Intrafamilial Variability from Childhood to Adulthood

**DOI:** 10.3390/genes17060722

**Published:** 2026-06-21

**Authors:** Massimiliano Chetta, Simone Lattarulo, Michele Stasi, Yevheniia Krylovska, Patrizia Lastella, Nicoletta Resta, Orazio Palumbo, Pietro Palumbo, Nenad Bukvic

**Affiliations:** 1 Laboratory di Biologia Molecolare UOC di Anatomia Patologica, Azienda Ospedaliero Universitaria San Giovanni di Dio Ruggi d’Aragona Scuola Medica Salernitana, 84121 Salerno, Italy; massimiliano.chetta@sangiovanniruggi.it; 2 Medical Genetics, Department of Precision and Regenerative Medicine and Ionian Area (DiMePRe-J), University of Bari Aldo Moro, 70124 Bari, Italy; lattarulo42@gmail.com (S.L.); michele.stasidoc@libero.it (M.S.); krilovskayevheniia@gmail.com (Y.K.); nicoletta.resta@uniba.it (N.R.); 3 Medical Genetics Section, University Hospital Consortium Corporation Polyclinics of Bari, 70124 Bari, Italy; patrizia.lastella76@gmail.com; 4 Division of Medical Genetics, Fondazione IRCCS Casa Sollievo della Sofferenza, 71013 San Giovanni Rotondo, Italy; o.palumbo@operapadrepio.it (O.P.); p.palumbo@operapadrepio.it (P.P.)

**Keywords:** POGZ, White–Sutton Syndrome—WHSUS, familial pediatric and adult cases

## Abstract

**Background/Objectives:** White–Sutton syndrome (WHSUS; OMIM 616364) is a rare neurodevelopmental disorder caused by pathogenic variants in the *POGZ* gene and characterized by developmental delay, intellectual disability, speech impairment, autism spectrum features, and dysmorphic traits. Although most reported cases are sporadic, inherited forms are exceptionally rare. We describe a familial case of WHSUS involving an affected mother and two children carrying a heterozygous *POGZ* nonsense variant, highlighting marked intra-familial phenotypic variability and expanding the clinical spectrum of the disorder. **Methods:** Clinical evaluation included multidisciplinary assessments. Genetic testing was performed using clinical exome sequencing (CES) with a virtual neurodevelopmental disorder (NDD) gene panel, followed by Sanger confirmation and segregation analysis in family members. The POGZ transcript reference NM_015100.3 was used for variant nomenclature and verified with the Mutalyzer tool. CNV detection from NGS data was performed using the Alissa CNV caller (Agilent) and visualized via IGV; the Xp11.22 microduplication was confirmed by chromosomal microarray (aCGH) and parental segregation analyses. **Results:** CES identified the heterozygous pathogenic *POGZ* variant c.1522C>T (p.Arg508*) in the female proband (III_6_), an infant presenting with global developmental delay, hypotonia, speech impairment, gait abnormalities, and characteristic dysmorphic features. Segregation analysis demonstrated maternal inheritance and confirmed the presence of the variant in her affected brother (III_4_), who also carries a de novo 1.79 kb microduplication at Xp11.22, while the maternal grandparents tested negative, indicating a de novo origin in the mother. The mother exhibited an attenuated phenotype, including mild neuropsychiatric and gastrointestinal manifestations. The variant is predicted to undergo nonsense-mediated decay (NMD), consistent with a moderate clinical presentation; however, experimental validation was not performed. **Conclusions:** This report documents a rare familial occurrence of WHSUS with highly variable expressivity. Our findings broaden the phenotypic and molecular characterization of *POGZ*-related disorders and emphasize the importance of comprehensive segregation studies and early genomic diagnosis. While experimental data link POGZ deficiency to DNA repair defects, no longitudinal clinical studies have demonstrated increased cancer risk in WHSUS; therefore, formal malignancy screening guidelines cannot be established at present, and this issue deserves future study in larger cohorts or registries.

## 1. Introduction

Neurodevelopmental disorders (NDDs) are a heterogeneous group of conditions with overlapping phenotypes, encompassing intellectual disability (ID), communication disorders, autism spectrum disorder (ASD), attention-deficit/hyperactivity disorder (ADHD), learning disorders, motor disorders, and epilepsy. They affect brain development and neurocognitive function from early childhood, leading to impairments in the cognitive, social, academic, and occupational domains [1,2,3].

Neuronal growth and differentiation begin during early embryonic development and progress through synapse formation in the first postnatal months. Consequently, impairments occurring in the early stages of development can alter brain architecture and compromise cerebral function throughout life [4,5]. NDDs are frequently characterized by incomplete penetrance and variable expressivity and are often inherited from asymptomatic parents [6].

Identifying the genetic causes of NDDs is crucial for understanding the molecular mechanisms underlying these disorders and for establishing genotype–phenotype correlations that may help monitor disease progression and anticipate potential complications [7]. Accordingly, differential diagnosis has become essential. The traditional phenotype-driven approach, based on the analysis of cohorts of individuals with shared clinical features, has increasingly been replaced by a “genotype-first” strategy, in which identification of the genetic etiology precedes and guides detailed clinical characterization of patient cohorts [8]. It has been estimated that 40–50% of complex NDD cases have a detectable genetic basis identifiable through genomic technologies such as chromosomal microarray (CMA), exome sequencing (ES), and whole-genome sequencing (WGS) [3,9].

Heterozygous pathogenic variants in the POGZ gene (TRANSPOSABLE ELEMENT-DERIVED PROTEIN WITH ZNF DOMAIN; OMIM 614787) have been identified as the genetic cause of POGZ-related intellectual disability syndrome, later termed White–Sutton syndrome (WHSUS; OMIM 616364), representing a paradigmatic example of the “genotype-first” approach [8]. WHSUS is an NDD classified among chromatinopathies and is characterized by developmental delay (DD), intellectual disability (ID), dysmorphic features, autistic traits, brain anomalies, and obesity. Reported dysmorphic features include microbrachycephaly, hypertelorism, flat malar ridge, midface hypoplasia, and prognathism [10,11].

POGZ is located on chromosome 1q21.3 and encodes the pogo transposable element with zinc finger domain, a multidomain protein containing a zinc finger cluster, an HP1-binding motif, and a transposase-derived DDE domain. It functions as a regulator of chromatin remodeling during chromosome segregation and mitotic progression through interaction with HP1α and activation of Aurora B Kinase, thereby promoting kinetochore assembly [12]. Gene expression peaks during embryonic development (8–9 weeks of gestation), gradually decreases toward birth, and remains at relatively low but stable levels throughout life [13]. POGZ is constitutively expressed in most tissues, with particularly high expression in the fetal and adult central nervous system (CNS), where it also acts as a transcriptional regulator within neuronal networks, including the cerebral cortex, hippocampus, basal ganglia, cerebellum, and spinal cord [13,14].

Most pathogenic variants of POGZ have been reported across nearly all exons, with the exception of exons 4–9 and 11 [8,13]. Furthermore, loss-of-function (LoF) variants have been shown to impair the proliferation and migration of neural stem cells during embryonic development [14].

**Figure 1:**
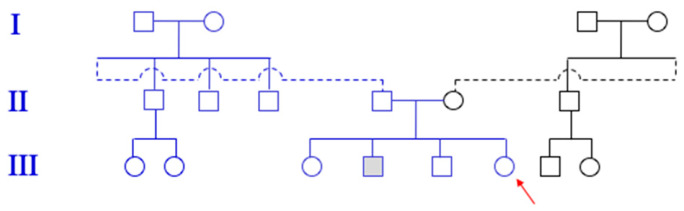
Pedigree depicted during the first visit.

Herein, we report on the case of an eleven-month-old infant (III_6_) referred to our genetic outpatient clinic for counseling due to a complex clinical presentation. The patient presented with global developmental delay, accompanied by distinctive dysmorphic features. The family history was notable for neurodevelopmental disorders (NDDs). The patient’s brother (III_4_) had intellectual disability and language delay associated with a de novo microduplication on chromosome Xp11.22, while her sister (III_3_) exhibited unilateral eyelid ptosis (Figure 1).

Clinical exome sequencing (CES) identified a heterozygous nonsense variant in the POGZ gene (c.1522C>T; p.Arg508*) in the patient, thereby establishing the diagnosis of White–Sutton syndrome (WHSUS). Given the limited number of cases reported in the literature, the patient’s phenotypic features were compared with those previously described. Segregation analysis demonstrated that the variant was of maternal origin; however, it was not detected in the maternal grandparents. This finding highlights a rare familial occurrence (affected mother and two children) that spans both pediatric and adult presentations with markedly different phenotypic expression. This observation adds to the small number of inherited POGZ cases reported to date and underscores the value of extended segregation analysis in mildly affected parents.

## 2. Materials and Methods

### 2.1. Patient Recruitment

The patient’s parents provided written informed consent to perform genetic testing, in accordance with the Declaration of Helsinki (1984) and its subsequent revisions, as well as any applicable local ethical and legal requirements.

### 2.2. NGS Panel for NDDs

Genetic analysis was performed using a massively parallel sequencing approach (Next Generation Sequencing, NGS) based on a multigene panel targeted for neurodevelopmental disorders. Genomic DNA was extracted from peripheral blood and collected in EDTA tubes using the EZ1 Advanced XL automatic extractor (Qiagen, Hilden, Germany) with the specific kit, following standard laboratory operating procedures. DNA concentration and purity were assessed by fluorimetry (Qubit, Invitrogen, Waltham, MA, USA) and spectrophotometry (NanoDrop 2000, Thermo Fisher Scientific, Wilmington, DE, USA). Genomic libraries were prepared using the SureSelect enrichment system (Agilent Technologies, Santa Clara, CA, USA), containing the coding sequences and adjacent introns of the genes included in the NDD panel. Library synthesis followed validated internal protocols, with indexing and hybrid capture. Library quality and quantity were verified using Agilent TapeStation and Qubit dsDNA Assay Kit. Massively parallel sequencing was performed on Illumina MiSeq (MiSeq Reagent Kit v3, 2 × 150 bp) and Illumina NextSeq 500/550 (Mid/High Output Kit v 2.5, 2 × 150 bp) platforms, based on the number of samples loaded per run. For the clinical case under examination, an average coverage of 249× was obtained, with a coverage of 100× in 96.95% of the target bases and 20× in 99.53% of target bases. The raw sequences (FASTQ) were subjected to bioinformatic analysis using Alissa Align & Call v 1.9 (Agilent) software for alignment and variant calling against the GRc37/hg19 reference genome. Filtering, annotation, and classification of variants were performed with Alissa Interpret v 5.2 (Agilent), applying maximum allele frequency thresholds of 0.00265 for autosomal dominant genes and 0.005 for autosomal recessive genes. The consulted databases included dbSNP, gnomAD, ClinVar, OMIM, and HGMD Professional. Variant nomenclature was verified using the Mutalyzer tool 3.2.0.dev03.2.0.dev0 (HGVS-compliant) and checked against the POGZ transcript NM_015100.3. Variant classification was conducted following the 2015 ACMG-AMP guidelines, with the integration of specific ClinGen criteria when feasible. A formal ACMG/AMP classification summary for the POGZ c.1522C>T variant is provided in Table 1. Genotype–phenotype correlation was based on clinical data. Clinically relevant variants were confirmed by Sanger sequencing, and segregation analysis was performed on the parents. CNVs were detected from NGS data using the Alissa CNV caller (Agilent) and visualized via IGV; the Xp11.22 microduplication was confirmed by chromosomal microarray (aCGH) and parental segregation analysis.

## 3. Results

### Patient’s Clinical History

The female proband was born spontaneously at term (38 weeks), with APGAR scores of 6/8, birth weight of 3810 g, length of 49 cm, and head circumference of 34 cm. During the perinatal period, slight respiratory distress was reported, which rapidly resolved with free-flow oxygen. Immediately, axial hypotonia was observed. Feeding was characterized by maternal breastfeeding until six months, followed by a regular and well tolerated weaning. Psychomotor development: Head movement control was reached at nine months of age, while torso movement control was established between the ninth and tenth month; crawling on all fours was reached at ten months, while standing position was reached at twelve months and autonomous ambulation at eighteen months. Early babbling was noted, with initial vocalizations occurring at approximately eight months of age. Nevertheless, language development proceeded slowly, with reduced vocal output. At 6 years, speech remains significantly underdeveloped and frequently unintelligible, despite continuous speech-language intervention. Relational interest was always optimal, with stable eye contact and precocious social smile. The patient has been previously examined through a wide range of clinical as well as instrumental examinations following a multidisciplinary path: pediatric neurology, cardiology, nephrology and ophthalmology, in line with international guidelines for NDDs. Cranial ultrasound evidenced a moderate periventricular hyperintensity, suggestive of a tissue vulnerability, which warranted a further follow-up. Cerebral magnetic resonance (MR), with and without intravenous contrast medium, did not reveal any significant alterations of morphology and signal, excluding structural CNS malformations. Electroencephalogram, repeated several times, was without epileptoid activity or specific paroxysmal abnormalities. Otoacoustic emissions and Auditory Brainstem Response (ABR) examination were normal. Basic laboratory blood analyses (hepatic, renal, thyroid, and blood count) did not reveal pathologic alterations. Echocardiogram, abdominal and renal echography results were normal, as were ophthalmic evaluations.

The patient was referred for genetic counseling by the pediatrician due to dysmorphic features and global delay. The patient was the eleven-month-old, fourth-born child of non-consanguineous Italian parents.

**Figure 2:**
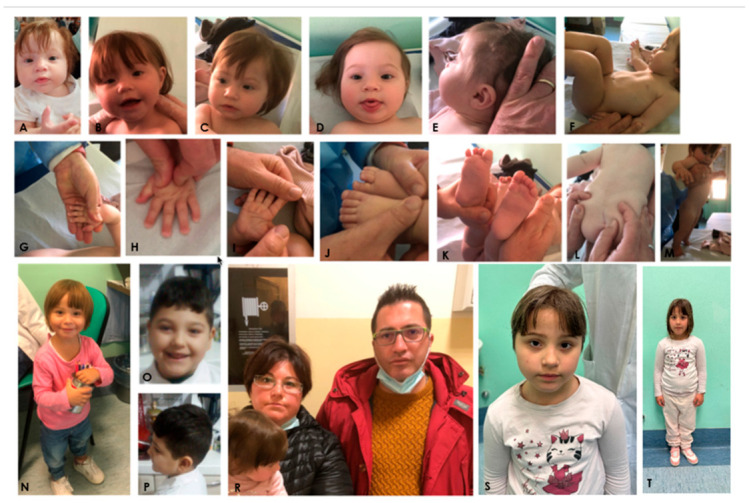
(**A**–**M**) Proband (III_6_) at 11 months old; (**N**) proband at 3 years; (**S**,**T**) proband at 6 years; (**O**,**P**) brother (III_4_) with de novo 1.79 kb microduplication at Xp11.22 (53,459,849_53,461,637)x2 and variant in the POGZ gene; (**R**) parents (mother II_5_ and father II_4_). Written informed consent was obtained for the publication of these identifiable images.

The objective examination at 11 months showed a peculiar facies: brachycephaly with relative microcephaly, high forehead, flat occiput, short neck, hypertelorism, upslanting palpebral fissures, sparse eyebrows (particularly in the medial part), telecanthus, depressed nasal bridge, open tented mouth, protrusion of the tongue, upper lip (Cupid’s bow), everted upper and lower lips, high palate, midface hypoplasia with chubby cheeks and apparently micro-retrognathia with a pointed chin. External ears were slightly posteriorly rotated with abnormally folded helices. The patient adapted positively to the environment, making eye contact and smiling socially. Simple vocalizations prevailed, and contextual commands were not always carried out. She was unable to stand alone, but moved independently from a sitting to a standing position, and maintained a sitting position independently with sufficient control of her trunk. During gait assessment, the 11-month-old infant did not assume a supported standing position. Instead, she consistently plantarflexed, bearing weight on her toes. When downward pressure was applied to encourage weight-bearing, she demonstrated withdrawal of the lower limbs, flexing them and avoiding full weight support. Measures at the time (11 months) were weight 6.7 kg (3rd percentile), height 68 cm (10th percentile), and head circumference 41.3 cm (2nd percentile; microcephaly) (see Figure 2A–M proband during the first visit, 2N proband at 3 years old, and Figure 2S,T proband at 6 years).

During the prenatal period, amniocentesis was performed with karyotype and array-CGH (aCGH) analyses, both with normal results: 46,XX; [arr (1-22,X)x2]. These analyses were performed because her brother (III_4_; Figure 2O,P) was affected by psychomotor delay, speech impairment, and dysmorphisms similar to those reported for the patient. He was found to carry a de novo 1.79 kb microduplication at Xp11.22 (53,459,849_53,461,637), initially identified during his sister’s pre-test consultation. This copy number variation (CNV) encompasses a portion of the HSD17B10 gene (17-beta-hydroxysteroid dehydrogenase X; OMIM 300256). The de novo origin of the variant was subsequently confirmed by segregation analysis of both parents; however, as the parental genomic results were not yet available at the time of prenatal testing for the proband, amniocentesis was performed. Both karyotype and comparative genomic hybridization (aCGH) analyses of our patient yielded normal results (46,XX; arr(1-22,X)x2).

Based on the patient’s medical history, physical examination, and clinical and laboratory findings, we proceeded with next-generation sequencing (NGS) using a neurodevelopmental disorders (NDD) panel.

This analysis identified a heterozygous variant in the POGZ gene (OMIM #614787), classified as pathogenic: c.1522C>T (p.Arg508*). This variant introduces a premature stop codon, resulting in a truncated protein.

**Figure 3:**
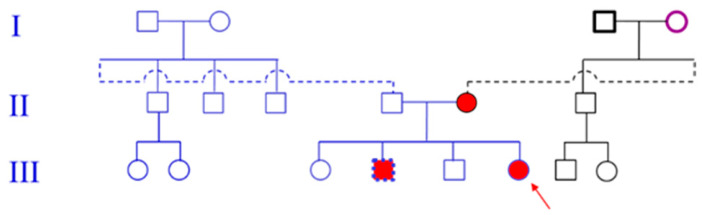
Final pedigree after implementation of molecular data: II_5_ carries the POGZ variant c.1522C>T; p.(Arg508*) in a heterozygous state, identified as a de novo variant. III_4_ carries a de novo Xp11.22 duplication (53,459,849–53,461,637) together with the POGZ variant c.1522C>T; p.(Arg508*) in a heterozygous state, which is maternally inherited. III_6_ carries the POGZ variant c.1522C>T; p.(Arg508*) in a heterozygous state, also maternally inherited.

Segregation analysis demonstrated that the c.1522C>T (p.Arg508*) variant in POGZ was maternally (II_5_) inherited and was also present in her brother (III_4_), while it was absent in the other siblings (III_3_, III_5_) and the father (II_4_; see pedigree, Figure 2R and Figure 3).

Because the mother exhibited a very attenuated phenotype of WHSUS and the variant had been reclassified from pathogenic to likely pathogenic, we extended the segregation analysis to the maternal grandparents (I3 and I4), both of whom showed no signs of WHSUS. This analysis excluded their carrier status, establishing a de novo origin of the variant in the mother and supporting its final reclassification as pathogenic (class 5, as summarized in Table 1).

Subsequently, additional genetic counseling was conducted with the mother. During this evaluation, she reported a tendency toward social withdrawal, difficulties with organization, heightened responses to stress, and recurrent episodes of vomiting. She also described a history of gastroesophageal reflux, constipation, and swallowing difficulties during childhood.

Subsequent follow-up of the proband revealed additional clinical features, including a wide-based gait and delayed language development. Detailed neuropsychological records and developmental history from her childhood were not systematically available; this represents a limitation of the current report.

## 4. Discussion

Since the initial description of White–Sutton syndrome (WHSUS) by White et al. in 2016 [8,10,11,13,15,16,17,18,19,20,21,22,23,24,25,26,27,28,29,30,31,32,33,34,35], more than one hundred cases have been reported, underscoring the rarity of this condition. Despite nearly a decade since its initial identification, the pathogenic mechanisms underlying WHSUS remain only partially elucidated, and its clinical expression is not yet fully characterized. This limited understanding continues to hinder diagnostic precision and the development of targeted therapeutic strategies, leaving WHSUS a largely enigmatic disorder. Moreover, only a small number of adult cases have been documented, and the long-term disease course remains poorly defined. In our study, which includes a rare familial observation (one adult and two pediatric cases), the condition appears generally stable and non-degenerative. Overall, the frequency and spectrum of clinical manifestations observed in our cohort are consistent with previous reports, confirming the marked phenotypic variability associated with this disorder.

The POGZ gene (OMIM 614787), located on chromosome 1q21.3, encodes a pogo transposable element–derived protein containing zinc finger domains. This protein interacts with the SP1 transcription factor and chromodomain helicase DNA-binding protein 4, and functions as a modulator of HP1α. Through these interactions, POGZ destabilizes HP1α–chromatin binding and plays a critical role in key meiotic and mitotic processes, including kinetochore assembly, sister chromatid cohesion, chromosome segregation, and mitotic progression [12]. Loss of POGZ function disrupts proper metaphase plate formation, leading to premature mitotic exit and resulting in genomic instability, polyploidy, increased apoptosis, and abnormal organ development [36,37]. During prenatal development, POGZ haploinsufficiency is thought to contribute to microcephaly by reducing the pool of neurogenic progenitor cells and, consequently, the total number of neurons [15]. Consistent with this, POGZ acts as a transcriptional regulator during forebrain development, promoting the expression of synaptic gene networks. Its expression persists postnatally across multiple regions of the central nervous system, including the cerebral cortex, hippocampus, pituitary gland, basal ganglia, cerebellum, and spinal cord [13,14,38].

**Figure 4:**
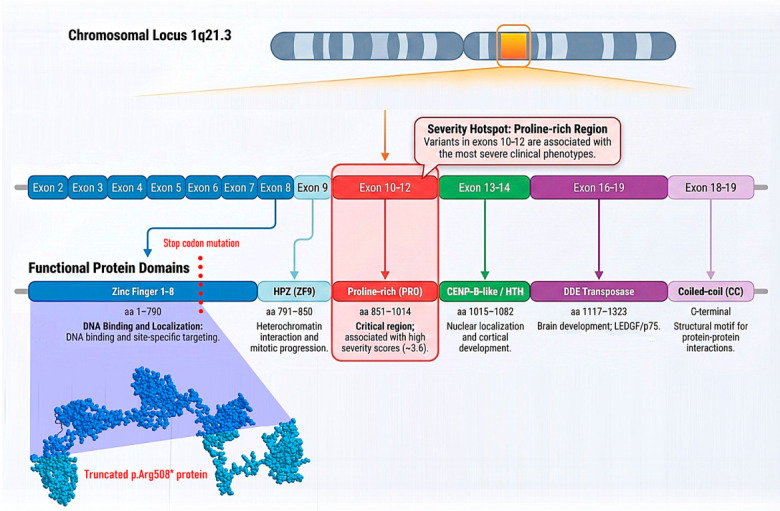
Schematic representation of the POGZ gene at chromosomal locus 1q21.3, showing how its exons assemble into a multi-domain protein. At the N-terminus, zinc fingers mediate DNA binding to anchor the protein at specific genomic sites. Furthermore, a CENP-B-like module helps organize nuclear architecture, a DDE transposase region supports brain development, and a coiled-coil tail serves as a docking hub for protein partnerships. The premature stop codon at p.Arg508* (red dotted line) results in premature protein truncation, leading to loss of all downstream C-terminal functional domains. Exons 10–12 (amino acids 851–1014) encode a proline-rich region that has a significant impact on clinical severity.

Pathogenic variants in the POGZ gene are associated with White–Sutton syndrome (WHSUS; OMIM 616364), a chromatinopathy characterized by intellectual disability, autism spectrum disorder, global developmental delay, speech impairment, and dysmorphic features. In our case, next-generation sequencing (NGS) targeting neurodevelopmental disorders identified a heterozygous nonsense variant in exon 9 of POGZ (c.1522C>T; p.Arg508*). This variant introduces a premature stop codon, replacing arginine at position 508 and resulting in a truncated protein within the C2H2 zinc finger domain (Figure 4).

Missense variants are generally associated with milder phenotypes [17,20,28,36,39], often limited to autism spectrum disorder or other neuropsychiatric features, whereas nonsense and frameshift variants tend to produce more severe clinical manifestations [6]. In the cohort of Nagy et al. [40], this trend was largely supported; however, exceptions have been observed, including a missense variant in the proline-rich region associated with increased clinical severity. These observations indicate that phenotypic outcome may depend less on variant class and more on factors such as variant location and susceptibility to nonsense-mediated decay (NMD). Variants predicted to undergo NMD are often associated with milder phenotypes, likely because degradation of aberrant transcripts results in haploinsufficiency while allowing partial compensation by residual normal mRNA. This mechanism may mitigate the severity of neurodevelopmental impairment and structural anomalies. Supporting this hypothesis, functional studies in Drosophila melanogaster have shown that knockdown of the POGZ ortholog primarily affects behavioral plasticity without causing major neurological defects [7]. In contrast, variants that escape NMD may produce truncated proteins with dominant-negative or gain-of-function effects, potentially interfering with the normal allele and leading to more severe phenotypes. Consistently, most pathogenic variants cluster in the C-terminal region of the protein. As reported by Nagy et al. [40], nonsense and frameshift variants subject to NMD, as well as missense variants located in the N-terminal half (including zinc finger domains 1–8 and the HPZ domain), are generally associated with milder phenotypes. Conversely, variants located within or near the proline-rich domain, particularly those escaping NMD, are associated with more severe clinical outcomes. Our findings are consistent with this framework. Application of the clinical severity scoring system proposed by Nagy et al. [40] yielded a score of 2 (moderate severity) for the proband, based on features observed up to six years of age, whereas the affected mother had a score of 1, corresponding to a mild phenotype.

### 4.1. Molecular Characterization of the p.Arg508* Variant and Functional Implications

The nonsense variant c.1522C>T introduces a premature stop codon within the C2H2 zinc finger region. According to established predictive criteria for nonsense-mediated mRNA decay (NMD), premature termination codons located more than 50–55 nucleotides upstream of the final exon–exon junction are generally expected to trigger NMD, whereas more C-terminal variants may escape this mechanism. Since residue 508 lies within the N-terminal portion of the protein, which is composed of 1410 amino acids, degradation of the mutant transcript through NMD is predicted [41,42].

This aspect is clinically relevant because studies in large patient cohorts have shown that nonsense and frameshift variants predicted to undergo NMD are often associated with comparatively milder phenotypes. In these cases, degradation of the aberrant mRNA primarily results in haploinsufficiency, thereby preserving partial function from the wild-type allele.

Nevertheless, the truncated p.Arg508* protein would retain the first eight zinc finger domains while completely lacking the C-terminal regions, including the remaining zinc fingers, the DDE endonuclease domain, and the interaction regions required for BRCA1/BARD1 complex recruitment [43]. This structural profile is particularly noteworthy: although the variant is predicted to be NMD-positive, any transcripts escaping degradation could potentially produce a truncated protein capable of competing with wild-type POGZ for HP1α binding, while remaining unable to mediate the essential C-terminal functions involved in DNA repair. Such a mechanism could theoretically result in a dominant-negative effect; however, this hypothesis remains speculative and requires experimental validation [40].

POGZ plays a critical role in the repair of DNA double-strand breaks (DSBs) through homologous recombination (HR). Functional studies have demonstrated that POGZ promotes the accumulation of BRCA1 and BARD1 at sites of DNA damage in an HP1-dependent manner. The p.Arg508* truncated protein, lacking the C-terminal region necessary for this recruitment, may therefore impair the DNA damage response. In cellular models depleted of POGZ, persistent γ-H2AX foci are observed at both 6 and 24 h after irradiation, indicating unresolved DSBs. Similarly, Pogz+/− haploinsufficient mice show spontaneous accumulation of DSBs in multiple tissues, including the brain and spleen, increased radiosensitivity, and impaired immunoglobulin class-switch recombination in B lymphocytes, leading to enhanced apoptosis secondary to unrepaired DNA damage [44].

### 4.2. Clinical Implications and Long-Term Surveillance

Despite substantial experimental evidence linking POGZ deficiency to genomic instability, no longitudinal clinical studies have yet demonstrated an increased cancer risk in individuals with White–Sutton syndrome (WHSUS). Nevertheless, emerging evidence suggests a potential role for POGZ dysregulation in tumorigenesis. In triple-negative breast cancer (TNBC), POGZ appears to have a dual function, promoting tumor growth while simultaneously suppressing metastatic behavior. Loss of POGZ enhances activation of the TGF-β signaling pathway, resulting in cytostatic effects but also increased mesenchymal transition and migratory capacity. Notably, the most aggressive TNBC subtypes show reduced POGZ expression, which has been associated with poorer clinical outcomes [44]. These experimental findings do not currently justify clinical cancer screening recommendations for WHSUS patients. Rather, this issue deserves future investigation in larger cohorts or disease registries before any formal surveillance guidelines can be established.

The proband’s brother (III_4_) also carries the familial POGZ variant but exhibits a markedly different clinical phenotype that may reflect a blended effect of both the familial POGZ variant and the unrelated de novo Xp11.22 microduplication. This 1.79 kb microduplication at Xp11.22 (chrX:53,459,849–53,461,637; GRCh37/hg19), which involves a portion of HSD17B10 (OMIM 300256), was classified as likely pathogenic under ACMG/ClinGen criteria based on confirmed de novo inheritance by parental segregation analysis (2A); absence from benign CNV databases (DGV gold standard, 3A); gene content implicating HSD17B10 in neurodevelopmental and metabolic disorders (4O); and overlap with an established genomic disorder region at Xp11.22 (5F) [45]. It is unclear whether the CNV alone is sufficient to explain the brother’s phenotype; therefore, a more cautious interpretation is that the brother may have a blended phenotype due to both the familial POGZ variant and the additional de novo CNV. This finding highlights the complexity of genetic counseling in families with neurodevelopmental disorders, where multiple contributing genetic factors may coexist. Particularly striking is the intra-familial variability observed between the mother’s mild and largely unrecognized phenotype and her daughter’s more overt clinical presentation. This contrast underscores the importance of comprehensive clinical evaluation and extended segregation analysis, without which both diagnosis and recurrence-risk counseling may be inaccurate or incomplete.

## 5. Conclusions

Taken together, these observations contribute to improved clinical recognition of POGZ-related disorders and strengthen genotype–phenotype correlations in a condition characterized by broad phenotypic heterogeneity. We also emphasize the importance of systematic long-term follow-up, as careful monitoring of phenotypic evolution over time remains essential for achieving diagnostic accuracy. Larger cohort studies and additional functional investigations will be necessary to further clarify the underlying biological mechanisms. At present, early diagnosis through clinical exome sequencing, combined with sustained multidisciplinary follow-up, represents the most effective strategy for optimizing clinical management and long-term outcomes for affected individuals and their families. While experimental models link POGZ loss to DNA repair defects, current clinical evidence is insufficient to recommend WHSUS-specific malignancy surveillance beyond standard population guidelines; this question should be addressed by future prospective studies.

## Figures and Tables

**Table 1:** ACMG/AMP criteria applied to POGZ c.1522C>T (p.Arg508*).

Criterion	Strength	Evidence
PVS1	Very Strong	Null variant in POGZ (established LoF mechanism)
PM2	Moderate	Absent from population databases
PS2	Strong	De novo occurrence in the mother
PP1	Supporting	Segregation with disease in affected relatives
PP4	Supporting	Phenotype highly specific for WHSUS

## Data Availability

The participants of this study did not give written consent for their data to be shared publicly; therefore, due to the sensitive nature of the research, supporting data are not available.
